# Queen Alexandra and King Edward Memorials

**Published:** 1912-07-27

**Authors:** James Gildea

**Affiliations:** 11 Hogarth Road, London, S.W.


### The Editor's Letter-Box.

### Queen Alexandra and King Edward Memorials.

*To the Editor of* The Hospital.

Sir,—You very kindly early in the year, in connection with the enclosed—" by desire of her Majesty Queen Alexandra, I have undertaken to collect particulars, for publication in due course, of all memorials of whatever description, at home, the Colonies, and abroad, to his late Majesty King Edward. I shall be much obliged, therefore, if those connected with the furtherance of such memorials will, as they are completed, kindly communicate with me, when I shall be glad to send particulars of the information required "—sent me a list in your issues of March 4 and April 19, 1911, of proposed memorials in hospitals, infirmaries, etc. May I venture to ask if you have published any subsequent list as the difficulties of finding out *where* these memorials have been initiated is very great?—Thanking you in anticipation,

Believe me, yours truly,

James Gildea.

11 Hogarth Road, London, S.W., July 15.

[No further list has been published, but we feel sure with this reminder hospital secretaries will co-operate with you in carrying out the wish of Queen Alexandra.—Ed. The Hospital.]

